# Dependency of mitochondrial quantity on blastocyst timeline obscures its actual effect to pregnancy outcomes

**DOI:** 10.3389/fendo.2024.1415865

**Published:** 2024-06-04

**Authors:** Tzu-Hsuan Chuang, Hsin-Hua Chou, Chin-Sheng Kuan, Shu-Cheng Liu, Chia-Wei Kao, Yi-Hsin Wu, Hsing-Hua Lai, Chia-Lin Hsieh, Yi-Ting Liang, Chien-Yu Chen, Shee-Uan Chen

**Affiliations:** ^1^ Stork Fertility Center, Stork Ladies Clinic, Hsinchu, Taiwan; ^2^ Graduate Institute of Clinical Medicine, National Taiwan University and College of Medicine, Taipei, Taiwan; ^3^ Department of Biomechatronics Engineering, National Taiwan University, Taipei, Taiwan; ^4^ Department of Obstetrics and Gynecology, National Taiwan University Hospital and College of Medicine, Taipei, Taiwan

**Keywords:** mitochondria, blastocyst timeline, morphokinetics, next-generation sequencing, single euploid embryo transfer

## Abstract

**Objectives:**

To explore the correlation between mitochondrial quantity and the blastocyst development timeline as well as their respective contributions to early pregnancy.

**Methods:**

A retrospective study was conducted using a dataset comprising 2,633 embryos that underwent preimplantation genetic testing for aneuploidy (PGT-A) between January 2016 and December 2023. The study was divided into three subsets to address distinct aspects: the representativeness of a single trophectoderm (TE) biopsy for mitochondrial quantity (n=43), the correlation between morphokinetic features and mitochondrial quantity (n=307), and the association analysis among mitochondrial quantity, blastocyst timeline factor, and reproductive outcomes (n=2,283). Distribution assessment of mitochondrial quantity across an individual blastocyst involved the identification within multiple biopsies and spent culture media. Timeline evaluation included correlating mitochondrial quantity with time-lapse datasets. Finally, multivariate logistic regression models, incorporating potential effectors alongside mitochondrial quantity, were employed to analyze their respective contributions to early pregnancy endpoints.

**Results:**

Of distribution assessment, mitochondrial quantity exhibited an even distribution across the entire trophectoderm (Spearman’s ρ=0.82), while no detectable mtDNAs in the corresponding spent culture media. Then the timeline correlation study revealed significant association between mitochondrial quantity and blastocyst features of both the day of expanded blastocyst formation (95% Confidence intervals, CIs: 0.27~4.89, p=0.03) and the timing of expanded blastocyst formation (tEB) (95% CIs: -0.24~-0.01, p=0.04) in the regression model, indicating a strong dependency between mitochondrial quantity and the blastocyst development timeline. For the contribution to early pregnancy, multivariate logistic regression models showed that the day of expanded blastocyst formation contributed to four endpoints persistently: positive for HCG (odd ratio, OR: 0.71, p=0.006), gestational sac (OR: 0.78, p=0.04), fetal heartbeat (OR: 0.71, p=0.004), and progression to 14 weeks (OR: 0.69, p=0.002). Contrastingly, no notable correlation was observed between the mitochondrial quantity and these endpoints.

**Conclusions:**

Strong interaction was observed between mitochondrial quantity and the blastocyst timeline, particularly the timing of expanded blastocyst formation. It suggests that the primary determinant influencing pregnancy outcomes lies in the time-dependent parameter of blastocyst rather than in the specific mitochondrial quantity.

## Introduction

Mitochondria, a highly dynamic organelle, play a pivotal role in the cellular energy supply and vital regulatory functions. Serving as the primary powerhouse, their quantity during oocyte maturation and early-stage embryo development follows a distinctive kinetic pattern finely tuned to meet biological demands (1). Intriguingly, the quantity of mitochondria undergoes noticeable changes throughout both oogenesis and embryogenesis. The primordial oocytes in mice contain around 10,000 copies of mitochondrial DNA (mtDNA) in the beginning of folliculogenesis (2). This number dramatically increases to between 50,000 to 550,000 copies by ovulation. In mature metaphase II (MII) oocytes, mitochondria play a crucial role as primary organelles, ready for fertilization (3).

Following fertilization, the quantity of mitochondria remains constant, and mtDNA replication halts during embryogenesis until implantation occurs (4–6). Previous reports have suggested that mitochondria in oocytes support early-stage embryo development before implantation. These mitochondria persistently distribute to dividing blastomeres, decreasing from approximately 80,000 copies per cell at the four-cell stage to 1,000 copies per cell at the blastocyst stage. Wai et al. (7) demonstrated a threshold of 40,000~50,000 per MII oocyte as the minimum requirement to bear the following development via the mouse model; while those with mtDNAs lower than this level failed to consecutively develop after implantation, though the blastocyst still can form with a reduced mtDNA level.

Despite the overall dynamic changes observed throughout both pre- and post- fertilization stages, mitochondrial quantity has been noted to exhibit considerable variability, differing even among disparate populations, even in cases where there is no known familial history of mitochondria-related diseases. Adhikari et al. (8) highlighted those factors such as aging, obesity, or polycystic ovarian syndrome (PCOS) could lead to mitochondrial dysfunction in oocytes, inducing the change of its quantity (9, 10). Several studies have also noted decreased mitochondrial quantity in women with advanced maternal age (AMA) and diminished ovarian reserve (DOR) (3, 11). More recently, Pasquariello et al. (12) elucidated those alterations in both the mitochondrial quantity and quality impact the oocyte competence in the aged females via mouse and human models. Therefore, several researchers have endeavored to establish mitochondrial quantity obtained from preimplantation genetic testing for aneuploidy (PGT-A) as a biomarker for embryo selection in IVF programs. Fragouli et al. (13, 14), Diez-Juan et al. (15) and Wells et al. (16) announced an increased mitochondrial quantity in euploid embryos with poor prognosis, suggesting its utility for predicting compromised reproductive outcomes. Nonetheless, conflicting findings from other investigators, such as Victor et al. (17), Treff et al. (18), and Scott et al. (19), consistently suggest no significant correlation between mitochondrial quantity and pregnancy results.

The contentious interpretations surrounding the application of mitochondrial quantity, commonly calculated as a mtDNA ratio (mtDNA content to nuclear DNA content) in embryo selection stem from a particular challenge. The validity of utilizing mitochondrial quantity derived from a single trophectoderm (TE) biopsy remains a subject of debate, which hinges on the question of whether such a biopsy accurately represents the mitochondrial quantity of the entire blastocyst, or of early-stage embryo development (20). Furthermore, while previous discussions have predominantly stratified mitochondrial quantity based on static embryo profiles such as blastocyst morphology, embryo gender, or maternal characteristics, the fidelity of a single mitochondrial quantity within dynamic characteristics of embryo remains inadequately investigated.

Accordingly, we hypothesized that the mitochondrial quantity at a single time point would interact with the specific embryo timeline, indicating that certain time-dependent parameters could serve as significant confounding factors when assessing the true impact of mitochondrial quantity on early pregnancy. The present study would concentrate on the representativeness of a single mitochondrial quantity within morphokinetic development of embryo, particularly the blastocyst stage. The primary analysis will delve into the distribution of mitochondria within a blastocyst. Subsequently, the second analysis aims to construct a linear regression model elucidating the interplay between mitochondrial quantity and time-dependent parameters. Upon validating its representational accuracy, the final analysis would conclude the significance of mitochondrial quantity in early pregnancy through multivariate logistic regression.

## Materials and methods

### Study design

The present study received approval from the independent Institutional Review Board (IRB, 202212143RINB) of the National Taiwan University Hospital (Taipei, Taiwan). Three key analyses were performed: firstly, assessing the distribution of mitochondrial quantity through multiple biopsies taken from the same blastocyst; secondly, analyzing the correlations between the morphokinetic parameters obtained from the time-lapse system and mitochondrial quantity; and finally, evaluating the significance of mitochondrial quantity in early pregnancy through multivariate logistic regression models. The entire workframe was depicted in **Supplementary Figure 1**.

### Study subjects

There were 43 surplus blastocysts utilized in the first analysis for assessing the distribution of mitochondrial quantity within the same blastocyst and culture environment. In the second analysis for time correlation, a total of 307 embryos were recruited to analyze the mitochondrial quantity and morphokinetic features. Then a large dataset was retrospectively compiled from 2,283 blastocysts obtained from the IVF couples undergoing single euploid embryo transfer (eSET), collected between January 2016 and December 2023, for the last associative study of pregnancy correlation. Written informed consents were secured from all the couples before participating in the programs. Personalized stimulation strategies were implemented (21–24).

### Embryo culture and biopsy

Embryos for the second analysis of time correlation were cultured at time-lapse incubators (Geri; Genea Biomedx, Sydney, NSW, Australia), applied with LGGG-050 medium (LifeGlobal; Coopersurgical, Ballerup, Denmark) under conditions of 37°C, 6% CO2, and 5% O2 (pH~7.4). A set of time-dependent videos was automatically recorded and determined by a well-trained clinical embryologist, including the time of pronuclei formation (tPNf), time of two-cell division (t2), time of four-cell division (t4), time of six-cell division (t6), time of eight-cell division (t8), time of morula formation (tM), starting time of blastocoel cavity formation (tSB), time of blastocoel cavity filled up (tB), and time of expanded blastocyst formation (tEB). Upon formation of the expanded blastocyst (Day 4 to Day 7), embryos meeting specific criteria as with both the inner-cell mass (ICM) graded as above B and distinctly trophectoderm (TE) graded as above C, based on the Gardner’s system (25) would be biopsied for subsequent PGT-A and mitochondria quantification. Biopsied samples were stored in 0.2-mL PCR tubes containing 2.5 μL of 1x PBS/1% PVP solution (Cell Signaling Technologies, Danvers, MA, US; Sigma, St. Louis, MO, US) and maintained at −20°C for following NGS procedures.

### Mitochondria quantification via NGS

Relative mtDNA measurement via PGT-A/NGS was utilized for retrospective regression analyses, incorporating morphokinetics and pregnancy interactions. The clinical biopsies underwent a series of procedures including lysis, pre-amplification, amplification, and barcoding, using the master mix of the Ion SingleSeq kit (Thermo Fisher Scientific, Waltham, MA, USA). Following whole genome amplification (WGA), the amplified products were purified using AMPure XP beads (Beckman Coulter, Pasadena, CA, USA) and quantified with the high-sensitivity (HS) Assay Kit (Qubit, Life Technologies, Waltham, MA, USA). The purified library was diluted for templating on the Ion Chef automatic machine (Thermo Fisher Scientific) and sequenced on the Ion S5 system (Ion ReproSeq PGS Kits). The sequenced data underwent analysis through the Torrent Suite Software (Thermo Fisher Scientific) to generate bam files, while Ion Reporter Software was utilized to identify chromosomal copy number variations (Thermo Fisher Scientific). The detailed methodologies have been extensively outlined in our previously published article (26). During the above process concurrently, the mtDNAs were also amplified and then sequenced. The generated bam files from Reproseq PGS procedures (Thermo Fisher Scientific) were leveraged for computing self-developed mitochondria quantification. This involved a series of steps where the sequences underwent specific quality control (QC) metrics using the pysam platform (Hege A and Köster J, v0.15.0, a python module that makes it easy to manipulate mapped short read sequence data stored in sam/bam files: https://github.com/pysam-developers/pysam, Release 0.15.0) for filtering. Reads that were unmapped, duplicates, or exhibited low-quality scores, as well as those with poor alignment performance, were excluded. Since relative mitochondrial quantification necessitates a reliable denominator for normalizing the biopsied cell numbers, a selected interval on chromosome 6, known for its stable percentage of obtained sequences and the least aneuploid frequency among somatic chromosomes (27), was screened and then selected. The reads corresponding to mtDNA were normalized against those of this selected interval to calculate the final mtDNA ratio. For more comprehensive details of target screening, refer to our previous report (28).

### Mitochondria quantification via qPCR

A secondary qPCR platform was employed to validate mtDNA detection in spent culture media samples, utilizing the NovaQUANT Human Mitochondrial to Nuclear DNA Ratio Kit (Merck, Darmstadt, Germany). Two pairs of mitochondrial genes and nuclear genes were quantified for calculating the relative mtDNA ratios: ND1 and BECN1; ND6 and NEB. The two mitochondrial DNA genes, ND1 and ND6, encode the subunit of NADH dehydrogenase; and the other two nuclear DNA, single-copy genes, BECN1 and NEB, encode the highly conservative eukaryotic proteins, Beclin 1 and Nebulin. The BECN1 and NEB were used as denominators for normalizing the biopsied cell number. Both the manipulation and calculation procedures were based on the manufacturers’ user guide. Succinctly, the WGA products from the PGT-A/NGS procedures were serially diluted to 0.1 ng/μL with DNase free water. Equal volume of 2X RT 2 Fast SYBR Green Mastermix (Thermo Fisher Scientific) was mixed with a total of 1ng WGA products. Then 20μL of the mixed solution was transferred into a commercial qPCR plate separately coated with pre-aliquoted qPCR primers for individual ND1, ND6, BECN1, and NEB genes. The qPCR assay was performed using a thermal cycler (QuantStudio 3 Real Time PCR System; Thermo Fisher Scientific) with a program: 95°C for 1 min of incubation, then amplification by 40 cycles of 95°C for 15 sec and 64°C for 1 min. Regarding the calculation, resultant Cts (Cycle threshold value, Ct) obtained from the qPCR assay can generate an average of 2^ΔCt^ from ND1 to BECN1, and ND6 to NEB, mean (2ΔCt of ND1- BECN1, 2^ΔCt^ of ND6- NEB), for calculation of mtDNA ratio.

### Distribution of mitochondria within a blastocyst

To assess the distribution of mitochondria across a particular blastocyst, sequencing data generated from multiple biopsies of the same embryo were applied. A total of 87 biopsied samples from 29 tested blastocysts were recruited. These tested embryos were originally vitrified using the Cryotech Vitrification kit (Cryotech; Tokyo, Japan), and were thawed using a Cryotech Warming kit. Then they were cultured in the one-step human embryo culture media (Global; LifeGlobal, CooperSurgical, Trumbull, Connecticut, US) until fully expanded. Each single blastocyst was biopsied at the TE opposite to ICM (distant TE), the TE near to ICM (close TE), and the ICM itself (**Supplementary Figure 2**). The biopsied procedures were the same with that of clinical implementation, using shearing force to separate the fractions (29). The mitochondrial quantity was evaluated using the NGS pipeline, and the association of mitochondrial quantity among the three biopsied sites of blastocyst was analyzed using Spearman’s rank correlation test. The rho (ρ) value between different TEs, and TE to ICM, were calculated respectively.

### Distribution of mitochondria in culture environment

The study for detection of mtDNAs in the respective spent culture media involved the collection of 28 paired trophoblast biopsies from 14 tested blastocysts alongside their corresponding culture media drops of 18 μL (day 3 to day 5). Again, the biopsied procedures were the same with that of clinical implementation (29), while the media drops were meticulously transferred into the 0.2-ml PCR tubes. Both of the two sample types went through the WGA and library preparation procedures for NGS analysis to derive the mitochondrial quantity. A Mann-Whitney U test utilized for comparing mitochondrial quantities of two sample types.

### Feature selection for regression analysis

A total of 307 time-lapse datasets were collected for the associative study of mitochondrial quantity and embryo development timeline, including t2~t8, tM, tSB, tB, and tEB. Descriptive analyses were initially conducted to provide a cohort overview of the associative matrix between time-dependent parameters and mitochondrial quantity. Features showing potential correlations with mitochondrial quantity were then incorporated into a multivariate linear regression model, given the limited size of the input data. For the final associative study of mitochondrial quantity and reproductive outcomes, a large dataset comprising 2,283 eSET cycles was utilized. Mitochondrial quantity and the features of oocyte source, maternal age, endometrial thickness, day of expanded blastocyst formation, morphology profiling, and embryo gender were assessed. Four logistic regression models were established to evaluate the impact of mitochondrial quantity on early pregnancy endpoints, including serum β HCG test results after two weeks of cryotransfer, detection of gestational sac, detection of fetal heartbeat, and ongoing pregnancy at 14 weeks.

### Statistical methods

Descriptive statistics including mean, standard deviation, and proportions were utilized to summarize the clinical characteristics of the studied cohort. Two distinct regression analyses were established to elucidate the influence of mitochondrial quantity, one pertaining to embryo development timeline and the other focusing on early pregnancy outcomes. All analyses were executed using STATA software (v.17; StataCorp LLC, Texas, USA).

## Results

### Distribution of mitochondria within a blastocyst

To evaluate the representativeness of mitochondrial quantity derived from a single trophectoderm (TE) biopsy for the entire blastocyst, a total of 29 embryos were biopsied at TE close to ICM, TE far from ICM, and ICM itself. Among these, two ICM biopsies failed to yield amplification, resulting in 85 informative biopsies with mitochondrial quantity outcomes (as mtDNA ratios). The mean mtDNA ratios for TE close to ICM, TE far from ICM, and the ICM itself were 0.74, 0.82, and 1.00, respectively. Spearman’s ρ values among informative samples for TE close to ICM, TE far from ICM, and between the two TE sites were 0.57, 0.64, and 0.82, respectively. Notably, the mtDNA ratios derived from the two TE biopsied sites exhibited the highest correlation (Spearman’s ρ = 0.82), while the correlation between the routine biopsy site (Distant TE) and the ICM showed a moderate-to-strong correlation (Spearman’s ρ = 0.57). The results of the correlation analysis were displayed in **Table 1**.

**Table 1:** Distribution of mitochondria within a blastocyst.

Biopsy site	Close TE	Distant TE	ICM
Sample pair	29	27	27
Amplification failed	0	2	2
Mean age	34.48	34.07	34.48
Mean mtDNA ratio	0.74	0.82	1.00
Correlation	Close TE to ICM	Distant TE to ICM	Close TE to Distant TE
Matched pairs	27	27	29
Spearman’s ρ	0.57	0.64	0.82
Scatter plots	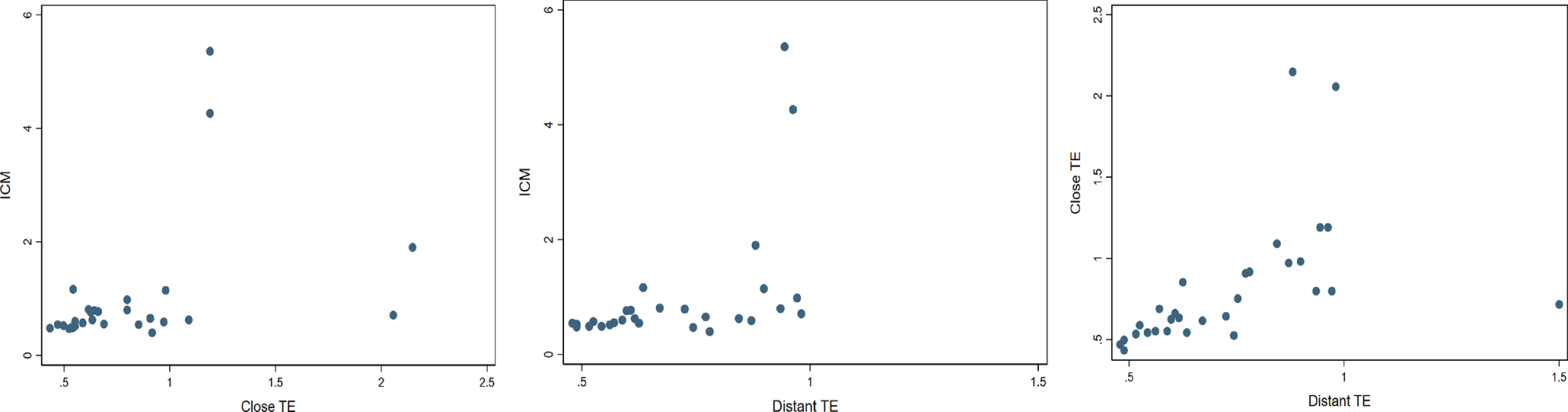		

### Distribution of mitochondria in culture environment

To evaluate the representativeness of mitochondrial quantity in the TE biopsies and their corresponding spent culture media, the paired samples were tested on both the NGS system (Ion S5; Thermo Fisher Scientific) and qPCR system (NovaQuant system, Merck). Through quantification via both the NGS and qPCR, No valid mtDNAs were detected in the spent media samples (~0), while an average mtDNA ratio of 2.69 was detected in their corresponding TE biopsies (**Table 2**).

**Table 2:** Distribution of mitochondria in culture environment.

	Paired	
Sample source	TE biopsy	Spent media
Sample number	14	14
Mean concentration of WGA (ng/ul)	24.71	20.02
Mean mtDNA ratio (NGS)	2.69	0.12
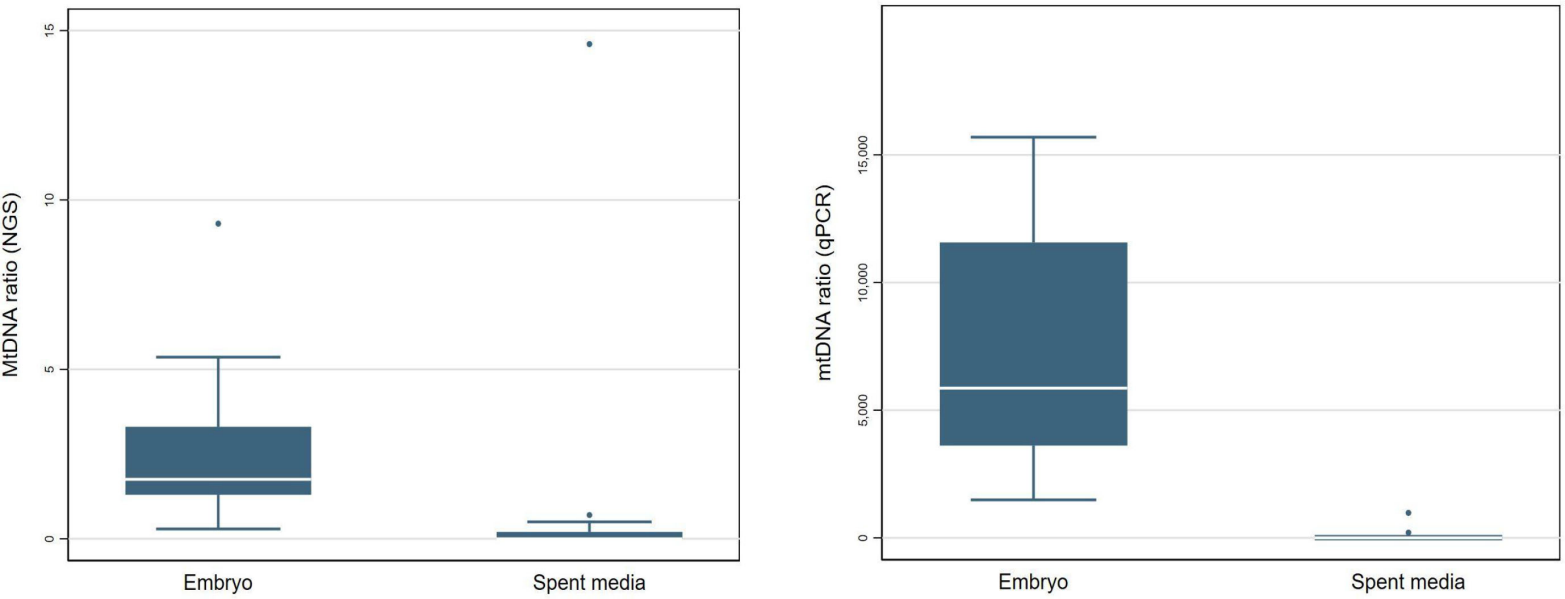		

### Correlation between mitochondrial quantity and embryo morphokinetics

A total of 307 embryos derived from 144 individuals were cultured at the time-lapse system, and the individual profile was illustrated in **Supplementary Table 1**, while the static embryo profile in **Supplementary Table 2**. Since the criteria for biopsy were strictly defined according to laboratory standards, specific preferences were observed in the distribution of certain blastocyst features: blastocyst formed on culture day 5, expansion score of 5, and ICM grading above class B, based on Gardner’s system.

**Figure 1:**
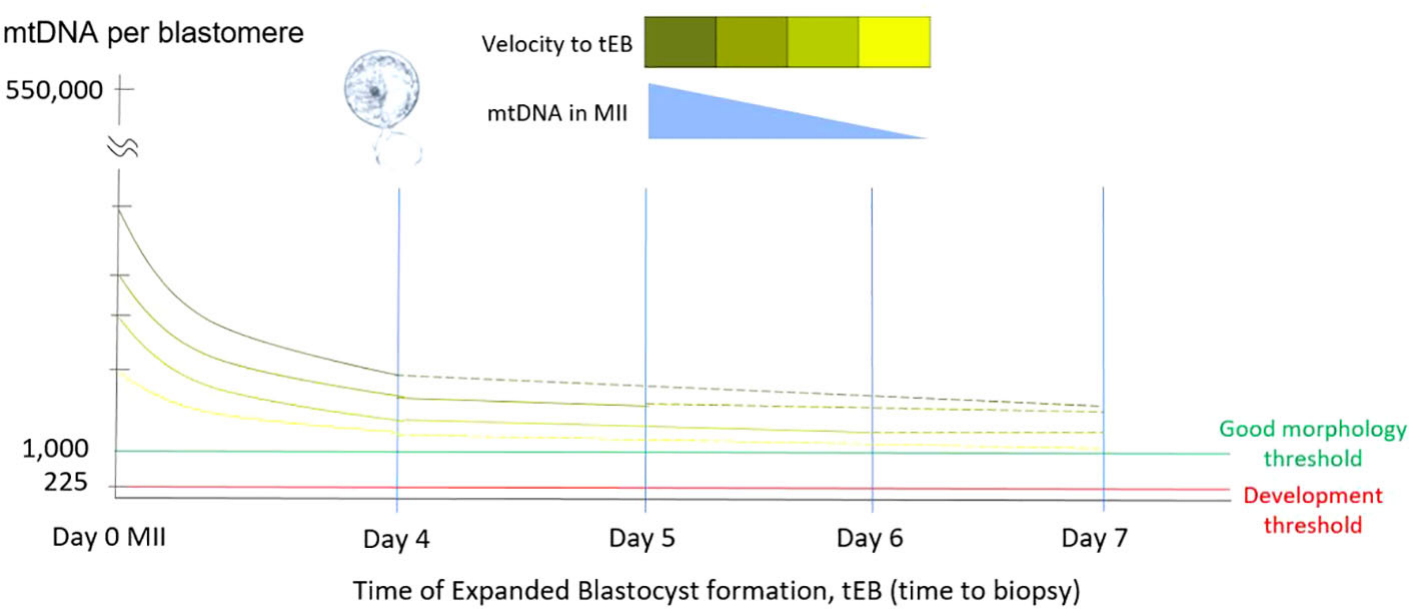
Relationship of mtDNA copy number and tEB. This diagram illustrates the relationship among the time of expanded blastocyst formation (tEB), as the timing of biopsy, the obtained mtDNA ratio, and the probable original mtDNA copy number of original MII oocyte. Since the precondition for biopsy has been defined by the operation laboratory, the obtained mtDNA ratio of a single biopsy shows negatively correlated with the tEB. Namely, faster the expanded blastocyst formed for biopsy (good morphology), higher the mtDNA involved in the original MII oocyte.

For preliminary preview of time-dependent features, **Figure 1** depicted a graph matrix illustrating the defined nine features and mitochondrial quantity obtained from TE biopsies. To simplify the backgrounds of input data, only the embryos with blastocyst formation day as 5, expansion score as 5, and gender as male were included in this analysis. Strong correlation were observed among nearer time points, such as t2-t4, t4-t6, tB-tSB, as determined by Spearman’s ρ exceeding 0.5; and moderate correlation was shown between the farther time points, such as t2-tB, t4-tB, t6-tM, and so on, with Spearman’s ρ ranging between 0.3 and 0.5. Of the morphokinetic features and mitochondrial quantity, merely weak correlation was obtained in t4, t6, tM, and tSB (Spearman’s ρ approximately 0.1~0.2).

### Regression analysis of mitochondrial quantity and morphokinetic features

Multivariate linear regression was applied to evaluate the morphokinetic features and mitochondrial quantity by adjusting the day of blastocyst formation, expansion state, and gender, which were identified as the influencers in our previous article (28). Due to data limitation, morphokinetic features exhibiting significant ρ values from the earlier Spearman tests of **Supplementary Figure 3** or showing relevance to the blastocyst timeline were retained in the multivariate linear regression model. As **Table 3** exhibited, the time of expanded blastocyst formation, tEB (as known as the time of biopsy) showed significant correlations with mitochondrial quantity. Upon analyzing the 95% confidence intervals (CIs), mitochondrial quantity exhibited a negative correlation with tEB. Furthermore, the interactions among the three significant features, day of expanded blastocyst formation, gender of embryo, and timing of expanded blastocyst formation, showed that they were independent of each other.

**Table 3:** Regression for mitochondrial quantity and morphokinetic features.

Involved embryo number	307	
Features	95% CI	p-value
Blastocyst formation (Day 4-Day 7)	0.27, 4.89	0.03*
Expansion score (Score 4-6)	-2.52, 0.61	0.23
Gender (Male, Female)	0.24, 1.71	0.01**
^a^t4	-.010, 0.18	0.57
^b^t6	-0.16, 0.03	0.24
^c^tM	-.027, 0.12	0.22
^d^tSB	-0.10, 0.14	0.73
^e^tB	-0.15, 0.06	0.41
^f^tEB (tBiopsy)	-0.24, -0.01	0.04*
Interaction		
Blastocyst formation_Gender	-0.25, 9.03	0.06
Blastocyst formation_tEB	-0.09, 0.04	0.44
Gender_tEB	-0.34,.05	0.14

a t4, time of 4-cell cleavage.

b t6, time of 6-cell cleavage.

c tM, time of morula.

d tSB, time of starting blastul.ation (blastocoel formation, tSB).

e tB, time of full blastocyst formation (blastocoel ≥ 1/2 embryo, tB).

f tEB, time of expanded blastocyst formation, namely time of biopsy.*p value <0.05, **p value <0.01.

### Regression analysis of reproductive outcomes and mtDNA ratios

At the final stage, we examined the correlation between reproductive outcomes and mitochondrial quantity in a substantial cohort, comprising 2,283 cryotransfer cycles involving euploid SET. Individual profile and stationary emrbyo information involved cryotransfer cycles was summarized in **Supplementary Tables 3**, **4**. Despite notable disparities in baseline characteristics and cycle profiles between individuals using self-oocytes and using donated oocytes, there was no discernible difference in the reproductive outcomes.

Then four early pregnancy endpoints were assessed: positive of HCG, detection of gestational sac, detection of fetal heartbeat, and ongoing pregnancy at 14 weeks. Employing multivariate logistic regression, we analyzed maternal variables, embryonic variables, and mitochondrial quantity in relation to these four endpoints, respectively, shown in **Table 4**. Eventually, day of blastocyst formation (odd ratio, OR: 0.69-0.71), endometrial (EM) thickness (OR: 1.06-1.10), and TE grading (OR: 1.36-1.46), consistently demonstrated effects on the four pregnancy endpoints, while maternal age, ICM grading, and gender of embryo exhibited marginal significance. However, no significant associations were observed between these reproductive endpoints and mitochondrial quantity. Additional analyses showed that remarkable interactions existed between the TE grading and embryo gender, TE grading and maternal age, respectively.

**Table 4:** Logistic regression analyses of mitochondrial quantity and four reproductive endpoints.

Outcome	HCG (+)^e^		Sac (+)^f^		FHB (+)^g^		14week (+)^h^	
Features	Odd ratio	p-value (95% CI)	Odd ratio	p-value (95% CI)	Odd ratio	p-value (95% CI)	Odd ratio	p-value (95% CI)
Oocyte source	1.37	0.10 (0.94, 1.98)	1.26	0.18 (0.89, 1.79)	1.22	0.24 (0.87, 1.72)	1.29	0.14 (0.92, 1.81)
Maternal age	0.97	0.06 (0.95, 1.00)	0.97	0.04* (0.95, 1.00)	0.98	0.06 (0.95, 1.00)	0.97	0.04* (0.95, 1.00)
^a^EM thickness	1.06	0.03* (1.00, 1.12)	1.10	0.001*** (1.04, 1.15)	1.08	0.001*** (1.03, 1.14)	1.09	0.001*** (1.04, 1.14)
^b^Day of eBT formation	0.71	0.006** (0.55, 0.91)	0.78	0.04* (0.61, 0.98)	0.71	0.004** (0.56, 0.89)	0.69	0.002** (0.54, 0.87)
Expansion state	0.77	0.28 (0.48, 1.24)	0.87	0.54 (0.55, 1.37)	0.88	0.58 (0.56, 1.39)	0.88	0.60 (0.56, 1.40)
^c^ICM grading	1.33	0.03* (1.02, 1.73)	1.26	0.06 (0.99, 1.60)	1.18	0.17 (0.93, 1.49)	1.10	0.42 (0.87, 1.38)
^d^TE grading	1.41	0.001*** (1.16, 1.72)	1.36	0.002** (1.12, 1.64)	1.37	0.001*** (1.14, 1.65)	1.46	0.001*** (1.21, 1.76)
Gender of embryo	1.22	0.04* (1.01, 1.47)	1.19	0.06 (1.00, 1.41)	1.13	0.16 (0.95, 1.35)	1.12	0.21 (0.94, 1.33)
mtDNA content	1.00	0.93 (0.96, 1.04)	1.00	0.87 (0.96, 1.04)	0.98	0.23 (0.94, 1.02)	0.98	0.42 (0.95, 1.02)
Interaction								
TE grading_ Gender	1.62	0.03* (1.05, 2.50)						
Age_TE grading			0.97	0.02* (0.94, 1.00)				

a EM, endometrium.

b Day of eBT formation, day of expanded blastocyst formation.

c ICM, inner-cell mass.

d TE, trophectoderm.

e HCG, human chorionic gonadotropin.

f Sac, gestational sac.

g FHB, fetal heartbeat.

h 14week(+), ongoing pregnancy at 14 weeks.*p value <0.05; **p value <0.01; ***p value <0.001.

## Discussion

The present study initially elucidated the relationship between a single mitochondrial quantity and the morphokinetic development of blastocyst, addressing analytical aspects such as: 1) representativeness of mitochondrial quantity from a single TE biopsy; 2) correlation between mitochondrial quantity and time-lapse features; and 3) association of mitochondrial quantity with early pregnancy. Our findings demonstrated the uniform distribution of mitochondria within the trophectoderm layer of a particular blastocyst. Subsequently, the results of temporal correlation revealed that mitochondrial quantity was closely tied to the actual timing of expanded blastocyst formation (as the timing of biopsy). Eventually, no significant association was observed between mitochondrial quantity and reproductive outcomes, including HCG positivity leading to ongoing pregnancy at 14 weeks, after adjusting for the day of expanded blastocyst formation.

The initial debate surrounding the application of mitochondrial quantity from a single TE biopsy in embryo selection during IVF practice concerned its representativeness to the entire blastocyst. Comparable concerns have been raised regarding the precision of PGT-A, particularly regarding confined ploidy mosaicism, which shows different chromosomal constitutions between the different TEs and ICM within an embryo, with an estimated incidence ranging from 15% to 30% (30, 31). Therefore, the present study aimed to assess the consistency of mitochondrial quantity detected within different biopsies of the same blastocyst. The findings corroborated a similar conclusion to our previous study (29): the consistency between different TE biopsies was higher compared to that between TE and ICM biopsies. It is reasonable to explain that the trophoblast lineage comprises the TE layer, which is responsible for implantation and future placental development, thus resulting in a uniform distribution of mitochondria within different TE biopsies. On the other hand, the epiblast/hypoblast lineages form the ICM for a distinct purpose, leading to a varied distribution of mitochondria compared to the TE biopsies (32).

Additionally, we conducted the detection of mitochondrial quantity in the spent culture media of blastocysts and corresponding TE biopsies. However, no mtDNAs were detected, suggesting that mitochondrial quantity may not be accessible for use in the non-invasive preimplantation genetic testing for aneuploidy (niPGT-A).

The second debate regarding the application of mitochondrial quantity centered on its reliance on the embryo timeline. Drawing from the fundamental work of Wai et al. (7), which established a threshold of 40,000 to 50,000 mtDNA copies per MII oocyte as the minimum requirement for subsequent fetogenesis in mouse models, it was observed that embryos with mitochondrial quantities below this threshold could progress to the blastocyst stage but fail to continue post-implantation development. In contrast, supporters of mitochondrial quantity as a potential selection marker announced that the reduced mitochondrial quantity leads to better reproductive outcomes (13–16), suggesting a compensatory response to intrinsic stress involved. Therefore, the present study utilized the time-lapse system to assess the interaction between the mitochondrial quantity at a specific time point and other morphokinetic features.

Our results indicated a significant negative correlation between mitochondrial quantity and the timing of expanded blastocyst formation (tEB, also as the timing of biopsy, tBiopsy). This finding is consistent with our previous study, which evaluated the correlation between the static feature, day of expanded blastocyst formation (day 4 to day 6) and mitochondrial quantity (28). Furthermore, the negative correlation observed between tEB and mitochondrial quantity emphasizes that the longer it takes to form an expanded blastocyst, the lower the original MII oocyte’s mitochondrial quantity may be contained (**Figure 1**).

In addition, the regression model also presented that besides timing of expanded blastocyst formation, the gender of the embryo stands out as the sole biological factor linked to the mitochondrial quantity. Bodri et al. (33) has uncovered that male blastocysts tend to exhibit faster expansion compared to female blastocysts in an investigation involving 505 expanded blastocysts, aligning with our additional post-hoc analysis showing a slight correlation between the gender of embryo and blastocyst formation.

Recently, De Munck et al. (34) and Bayram et al. (35) also applied the time-lapse system to investigate the mitochondrial quantity and morphokinetics. However, both the above approaches launched categorical cut-offs for either the mitochondrial quantity or embryo timeline, rather than using the original continuous values, and thus did not notice the high dependency between the two features. In our final analysis, four logistic regression models were utilized to explore the relationship between mitochondrial quantity and four pregnancy endpoints: HCG positivity, detection of gestational sac, detection of fetal heartbeat, and ongoing pregnancy up to 14 weeks. The day of expanded blastocyst formation, EM thickness, and grading of TE consistently demonstrated significance across all four endpoints. Furthermore, maternal age, grading of ICM, and embryo gender showed marginal significance for certain endpoints. After adjusting for other features, mitochondrial quantity did not influence any reproductive outcomes. Specifically, the contribution of mitochondrial quantity to pregnancy outcomes was relatively minor, indicating that the primary influencing factor is the time-dependent variable of blastocyst (day of expanded blastocyst formation, or timing of expanded blastocyst formation, tEB), which exhibits strong interaction with mitochondrial quantity, rather than mitochondrial quantity alone. Once more, this finding suggests that the previously observed effects of mitochondrial quantity on early pregnancy outcomes reported in published articles could potentially be confounded by the blastocyst timeline.

Nevertheless, the primary weakness of the study is its retrospective design, which the evaluation of relative mitochondrial quantity lacks comprehensive evidence concerning function and quality aspects, and the entire distribution of embryo characteristics was biased by the biopsy criteria of the operating laboratory. Conducting additional experiments on surplus embryos is imperative to better understand the time-dependent associations and provide enhanced interpretation of the current observations. To better evaluate the role of mitochondria more clearly, a randomized prospective trial comparing groups should be conducted in the future. Furthermore, the mtDNA tends to have a higher mutation frequency due to an insufficient repairing system and a higher reactive oxygen species (ROS) environment, and the effect of these heteroplasmic mitochondria involving *de novo* variants in early-stage embryos remains uncertain. Deep sequencing or other functional assays in cleavage and blastocyst biopsies may be useful to determine the role of mitochondria more extensively. On the practical side, application of machine learning-based technologies to analyze the true power of outcome prediction in mitochondrial parameters among specific populations is also worthy to investigate, since we only recruited general transfer features in the present logistic models.

## Conclusion

In conclusion, mitochondrial quantity, as derived from a single trophoblast biopsy, is notably influenced by the blastocyst timeline, particularly the timing of expanded blastocyst formation, signifying ongoing cell division progress. It showed no significant association with early pregnancy outcomes, and appears that the determinant influencer of pregnancy lies in the time-dependent parameter of blastocyst, rather than in the specific mitochondrial quantity.

## Data availability statement

The raw data supporting the conclusions of this article will be made available by the authors, without undue reservation.

## Ethics statement

The studies involving humans were approved by Institutional Review Board of the National Taiwan University Hospital (Taipei, Taiwan). The studies were conducted in accordance with the local legislation and institutional requirements. The participants provided their written informed consent to participate in this study.

## Author contributions

TC: Writing – review & editing, Writing – original draft, Visualization, Validation, Supervision, Software, Resources, Project administration, Methodology, Investigation, Funding acquisition, Formal analysis, Data curation, Conceptualization. HC: Writing – review & editing, Methodology, Investigation. CK: Writing – review & editing, Software, Project administration, Methodology. SL: Writing – review & editing, Software, Investigation, Formal analysis, Data curation. CK: Writing – review & editing, Investigation. YW: Writing – review & editing, Investigation. HL: Writing – review & editing, Resources, Project administration, Funding acquisition. CH: Writing – review & editing, Resources, Project administration, Funding acquisition. YL: Writing – review & editing, Visualization, Resources, Funding acquisition. CC: Writing – review & editing, Supervision, Investigation. SC: Writing – review & editing, Validation, Supervision, Investigation.
