# Supplementary material for: Dependency of mitochondrial quantity on blastocyst timeline obscures its actual effect to pregnancy outcomes
Source: Front Endocrinol (Lausanne). 2024 Jun 4;15:1415865. doi: 10.3389/fendo.2024.1415865 (PMC11182983; doi:10.3389/fendo.2024.1415865)
Supplement: Supplementary file 1 [file DataSheet_1.docx]

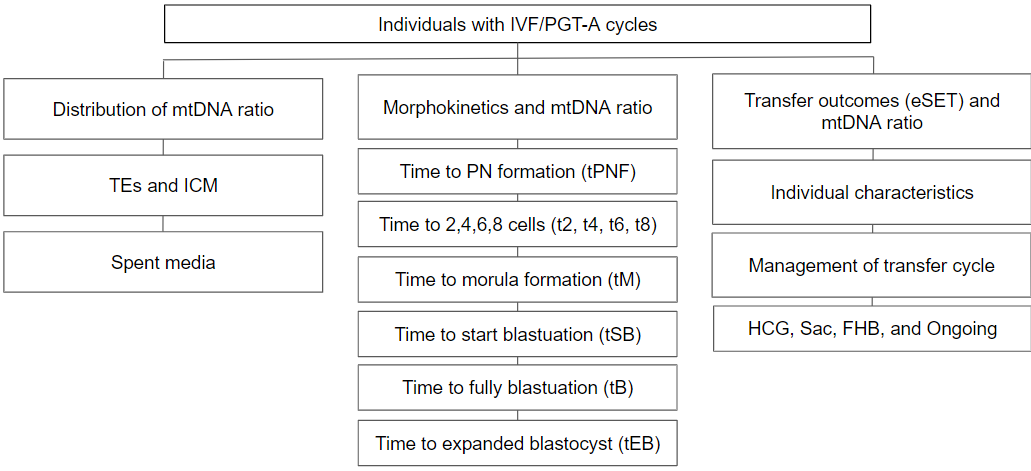


## **Supplementary Figure 1. Analytical workframe**

The analytical workframe is illustrated, encompassing three key analyses: 1) exploration of the distribution of mitochondrial quantity within the same blastocyst; 2) correlation of among the morphokinetic characteristics and mitochondrial quantity; 3) association between the early pregnancy endpoints and mitochondrial quantity.

mtDNA ratio (ratio of mitochondrial DNA to nuclear DNA, as mitochondrial quantity, as the mitochondrial quantity); PN (pronuclei); HCG (human chorionic gonadotropin); Sac (gestational sac); FHB (fetal heartbeat); Ongoing (continuous pregnancy until 14 weeks); and eSET (single euploid embryo transfer).


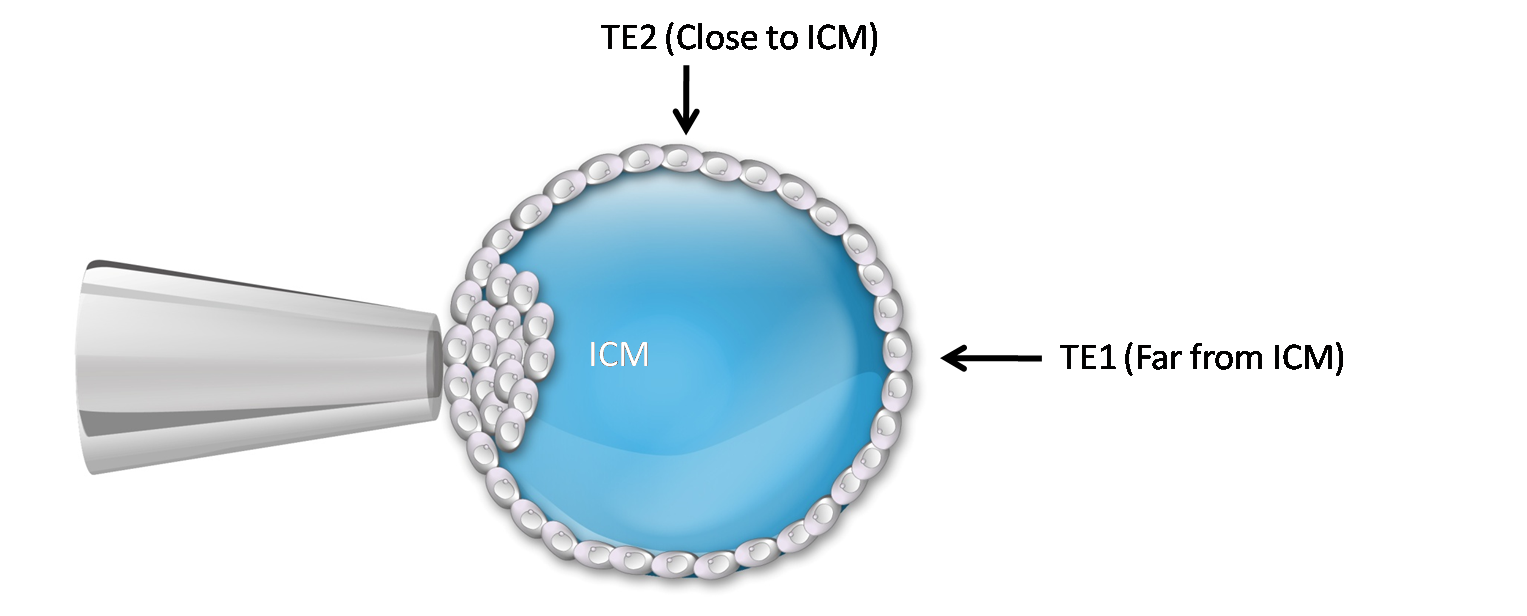


## **Supplementary Figure 2. Multiple biopsy within a blastocyst**

The ICM was held at the 9:00 position. One fraction was biopsied at the 3:00 position (TE1, far from the ICM), and the other fraction was biopsied at the 12:00 position (TE2, close to the ICM), and the last fraction was biopsied at the ICM. Each fraction contained 5-10 cells.


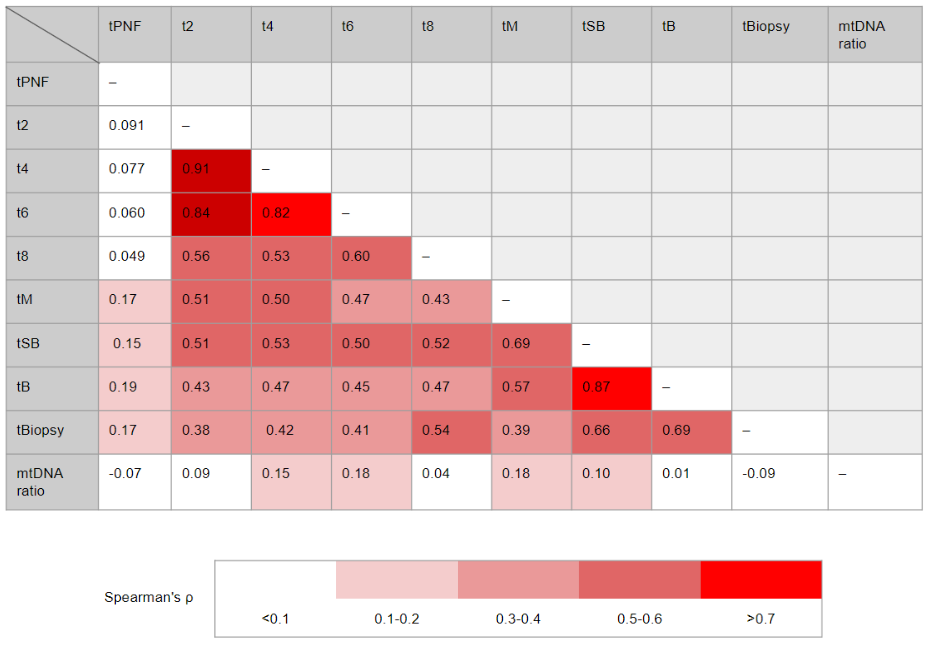


## **Supplementary Figure 3. Correlation matrix of mitochondrial quantity and time-dependent features**

The correlation among the nine features related to the morphokinetic timeline, including time points such as pronuclei formation (tPNF), 2-, 4-, 6-, and 8-cell cleavage times (t2~t8), morula formation (tM), starting blastulation (tSB), full blastocyst formation (tB), expanded blastocyst formation (tEB) as biopsy time, and mitochondrial quantity are presented. The strength of the correlation is represented as Spearman's ρ values in a heatmap format. The color gradient ranges from <0.1 to >0.7, where red color intensity signifies stronger correlation.
