# Supplementary material for: Dependency of mitochondrial quantity on blastocyst timeline obscures its actual effect to pregnancy outcomes
Source: Front Endocrinol (Lausanne). 2024 Jun 4;15:1415865. doi: 10.3389/fendo.2024.1415865 (PMC11182983; doi:10.3389/fendo.2024.1415865)
Supplement: Supplementary file 2 [file Table_1.docx]

##

## **Supplementary Table 1. Individual profile for time-lapse dataset**

|  | Time-lapse dataset |
| --- | --- |
| Number of involved IVF cycles | 307 |
| Individual profile (mean, SD) | |
| Number of involved IVF patients | 144 |
| Maternal age (years, SD) | 36.96 (5.36) |
| Body weight (kg, SD) | 55.68 (9.15) |
| Serum AMH^a^ value (ng/mL, SD) | 2.65 (2.02) |
| Antral follicle count (SD) | 7.24 (3.72) |
| COS^b^ profile (mean, SD) | |
| Final follicle count (SD) | 5.44 (5.43) |
| Leading follicle count (≥ 17mm, SD) | 0.86 (1.40) |
| Peak E2^c^ value (pg/mL, SE) | 2108.86 (10.69) |
| Peak LH^d^ value (mIU/mL, SE) | 10.36 (0.05) |
| Duration of COS (days, SD) | 10.72 (2.11) |
| Retrieved oocyte count (SD) | 13.42 |
| MII^e^ oocyte count (SD) | 10.50 |
| Maturation rate^f^ (%) | 83.86% |
| ^a^AMH, anti-müllerian hormone  ^b^COS, controlled ovarian stimulation  ^c^E2, estradiol  ^d^LH, luteinizing hormone  ^e^MII, metaphase II oocyte  ^f^Maturation rate, ratio of MII oocyte count to retrieved oocyte count | |

## **Supplementary Table 2. Static embryo profile for time-lapse dataset**

| Embryo number of time-lapse system: 307 | | | |
| --- | --- | --- | --- |
| Blastocyst formation [n (%)] | | Chromosome ploidy [n (%)] | |
| Day 5 | 220 (71.66) | Euploidy (%) | 114 (37.13) |
| Day 6 | 82 (26.71) | Mosaicism (%) | 52 (16.94) |
| Day 7 | 5 (1.63) | Aneuploidy (%) | 141 (45.93) |
| Expansion score [n (%)] | | Embryonic gender [n (%)] | |
| Score 4 | 4 (1.30) | Male (%) | 148 (49.01) |
| Score 5 | 288 (93.81) | Female (%) | 154 (50.99) |
| Score 6 | 15 (4.89) |  | (data missing: 5) |
| ICM^a^ grading [n (%)] | | ^a^ICM, inner-cell mass  ^b^TE, trophectoderm | |
| Grading A | 32 (10.42) |  |  |
| Grading B | 275 (89.58) |  |  |
| TE^b^ grading [n (%)] | |  |  |
| Grading A | 19 (6.19) |  |  |
| Grading B | 189 (61.56) |  |  |
| Grading C | 99 (32.25) |  |  |

#####

## **Supplementary Table 3. Individual profile derived from eSET**

|  | Self oocyte | Donated oocyte | p-value |
| --- | --- | --- | --- |
| Number of involved IVF cycles | 1417 | 866 | - |
| Individual profile (mean, SD) | | | |
| Number of involved IVF patients | 1220 | 742 | - |
| Number of failed IVF cycles | 0.43 (0.77) | 1.01 (1.46) | <0.0001 |
| Maternal age (years, SD) | 36.00 (3.66) | 24.26 (2.96) | <0.0001 |
| Body weight (kg, SD) | 56.86 (9.84) | 56.77 (8.94) | 0.68 |
| Body mass index (BMI, SD) | 22.02 (3.61) | 22.10 (3.43) | 0.66 |
| Serum AMH value^a^ (SD) | 3.64 (3.11) | 0.60 (0.71) | <0.0001 |
| FET^b^ profile (mean, SD) | | | |
| Baseline P4^c^ value (pg/mL, SD) | 0.17 (0.15) | 0.17 (0.15) | 0.99 |
| P4 value at cryotransfer (pg/mL, SD) | 43.35 (17.70) | 42.72 (18.81) | 0.91 |
| EM^d^ thickness at cryotransfer (mm, SD) | 9.44 (1.69) | 8.58 (1.59) | <0.0001 |
| Mean mtDNA ratio (SD) | 2.29 (2.37) | 1.60 (1.53) | <0.0001 |
| Reproductive outcomes | | | |
| HCG^e^ (+) rate (cycle) | 69.94 (991) | 69.63 (603) | 0.88 |
| Sac^f^ (+) rate (cycle) | 59.77 (847) | 61.09 (529) | 0.53 |
| FHB^g^ (+) rate (cycle) | 54.27 (769) | 56.24 (487) | 0.36 |
| Ongoing pregnancy rate ^h^ (cycle) | 51.52 (730) | 52.54 (455) | 0.64 |
| ^a^AMH, anti-müllerian hormone  ^b^FET, frozen embryo transfer  ^c^P4, progesterone  ^d^EM, endometrium  ^e^HCG, human chorionic gonadotropin  ^f^Sac, gestational sac  ^g^FHB, fetal heartbeat  ^h^Ongoing pregnancy, ongoing pregnancy at 14 weeks | | | |

###

## **Supplementary Table 4: Static embryo profile for eSET dataset**

| Embryo profile of eSET | | | |
| --- | --- | --- | --- |
| Blastocyst formation [n (%)] | | Chromosome ploidy [n (%)] | |
| Day 4 | 2 (0.09) | Euploidy (%) | 2283 (100) |
| Day 5 | 1948 (85.33) | ^a^ICM, inner-cell mass  ^b^TE, trophectoderm | |
| Day 6 | 328 (14.37) |  |  |
| Day 7 | 5 (0.22) |  |  |
| Expansion score [n (%)] | |  |  |
| Score 4 | 26 (1.14) |  |  |
| Score 5 | 2201 (96.41) |  |  |
| Score 6 | 56 (2.45) |  |  |
| ICM^e^ grading [n (%)] | |  |  |
| Grading A | 1914 (83.84) |  |  |
| Grading B | 369 (16.16) |  |  |
| TE^f^ grading [n (%)] | |  |  |
| Grading A | 256 (11.21) |  |  |
| Grading B | 1907 (83.53) |  |  |
| Grading C | 120 (5.26) |  |  |
